# Digital Health Technology Adoption Readiness Among Doctoral Nursing Students in Saudi Arabia: An Exploratory Qualitative Study

**DOI:** 10.3390/healthcare14111594

**Published:** 2026-06-05

**Authors:** Salha Salem Malki, Seham Mansour Alyousef

**Affiliations:** 1 College of Nursing, King Saud University, Riyadh 11451, Saudi Arabia; 2 Dammam Health Network, Eastern Health Cluster, Dammam 31433, Saudi Arabia; 3 Community and Psychiatric Department, Nursing College, King Saud University, Riyadh 12393, Saudi Arabia

**Keywords:** digital health, nursing, digital readiness, technology adoption, nursing education, Saudi Arabia

## Abstract

**Background**: Digital health technologies are increasingly integral to healthcare delivery worldwide; however, successful adoption depends on more than technological availability. In nursing, readiness is particularly important because digital systems increasingly shape documentation, communication, decision support, and care delivery. Within the context of Saudi Arabia’s healthcare transformation, doctoral nursing students are positioned as future educators, clinicians, and leaders whose perceptions can provide insight into digital health readiness and preparation. **Aim**: This study aimed to explore doctoral nursing students’ perceptions of their readiness to adopt digital health technologies in Saudi Arabia, guided by the Unified Theory of Acceptance and Use of Technology 2 (UTAUT2). **Methods**: This exploratory, qualitative, descriptive study recruited 9 doctoral nursing students from a public university in Saudi Arabia using purposive sampling based on predefined eligibility criteria. Individual semi-structured interviews were conducted online and audio-recorded. Data were analyzed using a hybrid inductive–deductive thematic approach. UTAUT2 informed the deductive component of the analysis, while inductive coding and cross-case comparison supported theme generation. **Results**: Four interrelated themes were identified. First, readiness was positive but conditional, shaped by movement from openness to professional necessity, familiarity, workflow fit, and caution about the possible weakening of foundational or manual competence. Second, adoption depended on practical value and system credibility, including access, convenience, efficiency, safety, documentation integrity, accuracy, privacy, and reliability. Third, adoption was organizationally mediated through leadership, peer culture, infrastructure, implementation conditions, training, follow-up, and academic preparation. Fourth, digital health was understood as supporting, not substituting for, nursing work by reducing avoidable burden and creating more space for direct care while preserving human presence, communication, and clinical judgment. **Conclusions**: In this sample of doctoral nursing students, digital health readiness was positive but conditional. The findings suggest that readiness reflects a context-sensitive professional judgment shaped by educational preparation, organizational support, system credibility, workflow compatibility, and the perceived ability of digital technologies to enhance nursing work rather than replace it. **Implications**: The findings suggest that nursing education and practice should strengthen applied digital health competencies through simulation-based preparation, electronic documentation training, privacy and ethics education, workflow-aligned implementation, and sustained organizational support.

## 1. Introduction

Healthcare systems worldwide have undergone rapid digital transformation over the last decade, with increasing emphasis on using digital technologies to make care more accessible, efficient, coordinated, and responsive to population needs [1,2]. Technologies such as electronic health records, telemedicine, remote monitoring systems, and AI-supported applications are increasingly used to support data flow, clinical communication, decision support, and patient engagement [1,2]. However, the benefits of these technologies are not achieved solely through technological availability; they depend on whether health professionals and organizations are digitally capable, adequately supported, and prepared to integrate digital tools into routine care [2,3,4].

Within this broader transformation, the WHO defines digital health as a field of knowledge and practice associated with the development and use of digital technologies to improve health [2]. The WHO further emphasizes that digital health can generate value when implementation is supported by appropriate governance, workforce capability, planning, and a coordinated system design [2]. OECD analyses indicate that many health systems remain insufficiently prepared to realize the full benefits of digital transformation, partly because data environments, governance arrangements, and technical infrastructures are fragmented, outdated, or insufficiently interoperable [1]. At the level of professional practice, this shifts attention to readiness for adoption, sustained use, and context-sensitive integration into everyday work [2,3].

This focus on readiness is particularly important in nursing because nurses routinely engage with electronic documentation, technology-enabled communication, monitoring systems, telehealth, and other information-intensive care processes [5,6,7]. Existing reviews suggest that digital health adoption in nursing can improve access to information, workflow efficiency, and patient care; however, these benefits depend on supportive implementation conditions, including adequate infrastructure, manageable workload, user proficiency, workflow integration, training, perceived usefulness, intuitive systems, leadership, local champions, and organizational investment [3,5,7]. Taken together, this evidence frames digital health adoption in nursing as a socio-technical process rather than a simple matter of individual willingness to use technology.

Building on this sociotechnical perspective, readiness should not be inferred from exposure to technology or digital knowledge. Longhini et al. [8] found that digital health competence among nurses and healthcare professionals was generally moderate, with actual use of digital technologies weaker than knowledge about them. Educational evidence further suggests that digital competence can be strengthened through multimethod, practical, and interactive training, supported by organizational encouragement and needs-based guidance [9]. However, continuing education for nursing digital competencies remains underdeveloped, with much of the literature still focused on frameworks or curricular recommendations rather than on clearly evaluated programs [10]. Together, this evidence underscores the importance of educational preparation, repeated practice, and organizational support in helping healthcare professionals move from general digital knowledge to confident application in practice settings.

In addition to competence development, the influence of organizational leadership and implementation climate is crucial in determining whether digital readiness is effectively translated into sustained nursing practice. Burgess and Honey [11] identified nurse leaders as pivotal facilitators of adoption by bridging the digital and clinical domains, supporting the development of digital practices, maintaining a presence in clinical settings, and advocating for nurses’ involvement in implementation processes. At a broader policy and systemic level, Janes et al. [12] observed that nurses and midwives contribute to digital health policy and practice; however, their contributions remain underutilized and insufficiently supported by the system. Evidence from the use of telehealth and mobile technology corroborates this perspective, indicating that successful digital adoption is influenced by factors such as usability, workflow integration, communication requirements, privacy, infrastructure, and organizational support [13,14,15]. These studies redirect the focus from individual preparedness to implementation environments that render digital practice usable, legitimate, and sustainable.

In light of the international evidence base, Saudi Arabia presents a pertinent national context for assessing digital health readiness. Under the auspices of Vision 2030, the Health Sector Transformation Program aims to restructure the health sector into an efficient and integrated system. Its strategic objectives include facilitating access to health services, enhancing service quality and efficiency, promoting prevention, and strengthening integrated care [16]. Within the healthcare domain, empirical evidence from Saudi Arabia indicates a strong intention among healthcare practitioners to adopt telehealth, with performance expectancy and social influence identified as significant predictors of this intention [17]. These national priorities and emerging empirical findings highlight the need to examine how digital health readiness is conceptualized in nursing education and professional preparation.

Although digital health readiness, digital health education, and technology acceptance have been examined among healthcare professionals, practicing nurses, and nursing students [11,18,19], the perspectives of doctoral nursing students have not been sufficiently examined. This is an important gap because doctoral nursing students occupy a distinctive position at the intersection of advanced academic preparation, professional experience, research engagement, and potential future educational and leadership roles. Their perspectives can illuminate readiness as more than individual willingness or intention to use technology; rather, it may be understood as a professional, context-sensitive judgment shaped by educational preparation, system credibility, organizational support, and the perceived fit between digital technologies and nursing work. By focusing on doctoral nursing students in Saudi Arabia, this study provides a qualitative account of digital health technology adoption readiness within the context of Saudi Arabia’s healthcare transformation and extends technology acceptance perspectives by foregrounding the professional, contextual, and organizational dimensions of readiness. These considerations align closely with the Unified Theory of Acceptance and Use of Technology 2 (UTAUT2). UTAUT2 explains technology acceptance and use through constructs relevant to digital health readiness, including performance expectancy, effort expectancy, social influence, facilitating conditions, habit, price value, and hedonic motivation [20]. In this study, UTAUT2 was not used to test predefined relationships but to guide attention to how participants described usefulness, ease of use, social and organizational influence, repeated experience, and practical value. This approach is consistent with digital health evaluation literature, which emphasizes the value of theory-informed and context-sensitive frameworks for understanding real-world implementation [21]. Accordingly, UTAUT2 provides a flexible theoretical lens for exploring readiness as a contextually shaped and professionally situated phenomenon among doctoral nursing students.

Therefore, this exploratory qualitative study aimed to explore doctoral nursing students’ perceptions of readiness for digital health technology adoption in Saudi Arabia, with UTAUT2 used to guide the inquiry. Specifically, the study sought to describe doctoral nursing students’ perceptions of digital health readiness and to identify the perceived facilitators, barriers, and organizational conditions shaping this readiness. The guiding research question was: How do doctoral nursing students perceive readiness for digital health technology adoption in nursing, and what factors shape this readiness?

## 2. Materials and Methods

### 2.1. Study Design

This study employed an exploratory qualitative descriptive design to explore doctoral nursing students’ perceptions of readiness for digital health technology adoption. A qualitative descriptive approach was appropriate because the study aimed to produce a practice-oriented account of participants’ views on readiness, perceived facilitators, barriers, and contextual conditions affecting digital health adoption, rather than to develop a formal explanatory theory [22,23]. The study was conceptually guided by the Unified Theory of Acceptance and Use of Technology 2 (UTAUT2), which shaped the development of the interview guide and served as a sensitizing framework during analysis rather than as a rigid analytic template [20]. Reporting was informed by the Consolidated Criteria for Reporting Qualitative Research (COREQ), where applicable, to enhance transparency in describing the study context, recruitment, data collection, analysis, the researcher’s role, and strategies used to support methodological rigor [24] (Supplementary Materials, File S1).

### 2.2. Study Setting and Participants

The study was conducted within a doctoral nursing program at a public university in Saudi Arabia. The target population comprised doctoral nursing students enrolled in the selected program during the recruitment period. Purposive sampling with voluntary participation was used because the study required information-rich participants who could reflect on digital health readiness from advanced academic and professional perspectives.

The first author invited students to participate through the program’s established student communication channel. The invitation was shared with all eligible doctoral nursing students in the selected program during the recruitment period (n = 20). A reminder was sent on the same channel a few days later. Nine students completed individual interviews, whereas 11 did not respond. No student who responded was excluded based on the eligibility criteria, and no participant withdrew after providing consent. Reasons for non-response were not collected.

To minimize perceived obligation, the invitation stated that participation was voluntary, non-participation would have no academic or professional consequences, and interested students could contact the researcher directly. Although the first author was situated in the same academic context as the participants, she had no teaching, supervisory, or evaluative authority over them.

The inclusion criteria were as follows:

▪ Current enrollment as a doctoral nursing student in the selected nursing doctoral program during the recruitment period
▪ Prior academic or professional nursing background relevant to discussing digital health technology adoption in nursing

The exclusion criteria were as follows:

▪ Enrollment in an undergraduate, master’s, or non-nursing doctoral program
▪ Having a direct teaching, supervisory, or evaluative relationship with the interviewer

Written informed consent was obtained from all participants before the interviews. Nine doctoral nursing students participated in the study, and their characteristics are summarized in Table 1.

Sample adequacy was assessed using the principles of information power and thematic sufficiency rather than statistical representativeness [22,25]. The study’s aim was focused, the sample was specific, and participants had relevant academic and professional perspectives on digital health technology adoption in nursing. During data collection and preliminary analysis, transcripts, analytic memos, and the cross-case matrix were reviewed iteratively. In later interviews, participants’ accounts repeated patterns identified in earlier interviews, particularly regarding workflow fit, system credibility, organizational support, training, privacy, and the supportive rather than substitutive role of digital health. No substantially new categories were identified. Therefore, no further reminders were sent, and the dataset was considered sufficient to address the research question and develop coherent themes. The small purposive sample and the absence of data on reasons for non-response are acknowledged in the Study Limitations section.

### 2.3. Data Collection

Data were collected by the first author through individual semi-structured interviews conducted remotely via audio-only Zoom (Zoom Video Communications, Inc., San Jose, CA, USA) between 1 and 14 March 2026. The interviews were conducted in Arabic, completed in a single session, and lasted approximately 12–26 min, with a mean duration of approximately 19 min. No non-participants were present during the interviews. Although the interviews were relatively brief, the interview guide was structured around five open-ended questions aligned with the study aim and UTAUT2-informed inquiry. Follow-up probes were used to clarify participants’ responses and elicit examples of readiness, workflow fit, system credibility, privacy, training, organizational support, and perceived role of digital health in nursing work. Written informed consent was obtained before participation, and verbal confirmation of consent was obtained before the audio recording commenced. Participants were informed that participation was voluntary and that they could withdraw from the study at any time without penalty.

The interview guide was developed specifically for this study and informed by the UTAUT2 framework [20] (Supplementary Materials, File S2). The interview included five open-ended questions exploring readiness to adopt digital health technologies, factors encouraging adoption, perceived barriers, organizational influences, and the potential contribution of digital health to future nursing practice. Follow-up probes were used to clarify participants’ responses and elicit examples related to workflow fit, system credibility, privacy, training, organizational support, and the perceived role of digital health in nursing. Brief demographic prompts regarding age, gender, and academic and/or professional experience were also included. The first interview was treated as a pilot interview to assess the clarity and relevance of the interview guide. As no substantive revisions were required, this interview was retained in the final dataset. All interviews were audio-recorded with participants’ permission and transcribed verbatim in Arabic by the first author. Brief field notes were used to support reflexivity and early analytic thinking, but were not treated as a separate dataset. The participant quotations selected for presentation in the manuscript were translated from the Arabic originals into English at the reporting stage.

### 2.4. Data Analysis

Data were analyzed using a hybrid inductive–deductive thematic analysis, informed by thematic analysis principles and approaches that combine theoretically informed coding with inductive theme development [26,27]. Deductive coding was informed by UTAUT2 and guided attention to adoption-related concepts, including performance expectancy, effort expectancy, social influence, facilitating conditions, experience, and habit [20]. Inductive coding allowed additional meanings to emerge from participants’ accounts, including system credibility, workflow fit, organizational mediation, foundational competence, and the professional meaning of digital health in nursing. The final themes were not organized as predefined UTAUT2 categories; rather, they were developed through iterative comparison of inductive and deductive codes.

To make the theoretical integration more explicit, a UTAUT2 mapping table was developed to show how the selected UTAUT2 constructs informed the interview guide, deductive coding, and the development of the final themes (Supplementary Materials, File S2). Constructs such as price value and hedonic motivation were less prominent because participants discussed digital health primarily as academic and clinical users within institutional systems rather than as purchasers of technologies or as users motivated by enjoyment.

The analysis was first-author-led, iterative, and recursive. The first author read the Arabic transcripts repeatedly, manually coded them in Microsoft Excel (Microsoft Corporation, Redmond, WA, USA), and organized the data using coding matrices and an evolving coding book. Analytic memos documented emerging interpretations, methodological reflections, and analytic decisions. Codes were compared within and across interviews, then clustered into candidate themes and subthemes, reviewed against the full dataset, refined, and clearly defined and named. Cross-case comparison was used to examine convergence, variation, and accounts that confirmed, qualified, or complicated emerging interpretations. These procedures were consistent with qualitative data analysis strategies involving coding, memoing, data display, cross-case comparison, and theme development [22,28]. The Arabic transcripts remained the primary source texts for interpretation, while final themes and subthemes were developed in English for manuscript reporting. Selected quotations translated into English were checked against the Arabic transcripts by the first author to preserve meaning.

### 2.5. Trustworthiness and Reflexivity

Trustworthiness was supported through strategies that addressed credibility, dependability, confirmability, and transferability [29]. Credibility was strengthened through a pilot interview, audio-recorded interviews, verbatim transcription, repeated engagement with the transcripts, iterative comparison across interviews, and the use of illustrative quotations to link participants’ accounts to the final themes. Dependability and confirmability were supported through an evolving codebook, analytic memoing, and a documented analytic trail of coding decisions and theme development. Transferability was supported by providing clear descriptions of the study context, participants, sampling approach, data collection, and analytical procedures. At the time of the study, the first author, who conducted the interviews and led the analysis, was a female nursing administration director with clinical and administrative nursing experience and was enrolled in a Doctor of Philosophy in Nursing program.

Reflexivity was addressed by acknowledging this insider position within the same academic context as the participants. This position may have facilitated rapport and shared understanding; however, it also required attention to how familiarity with the setting could influence recruitment, questioning, coding, interpretation, and the participants’ responses. To minimize perceived obligation, the first author had no teaching, supervisory, or evaluative authority over the participants, and recruitment emphasized voluntary participation. Reflexive memos were used after the interviews and during the analysis to document the researcher’s positioning, methodological reflections, and analytic decisions. Participant member checking of transcripts or themes was not conducted; instead, credibility and confirmability were supported through verbatim transcription, repeated transcript review, analytic memoing, cross-case matrix comparison, illustrative quotations, and an audit trail of analytic decisions.

### 2.6. Ethical Considerations

Ethical approval was obtained from the relevant institutional research ethics committee before data collection commenced. Written informed consent was obtained before participation, and verbal confirmation of consent was obtained again before the audio recording commenced. Participants were informed of the study’s purpose, the voluntary nature of participation, the use of audio recordings for research purposes only, and their right to decline participation or withdraw at any time without consequence. Confidentiality was maintained throughout the study period. Identifying information was removed from the transcripts, and participants were assigned codes (P01–P09) for analysis and reporting. The setting details and participant characteristics were reported at an aggregate level, where possible, to reduce the risk of deductive identification.

## 3. Results

### 3.1. Participant Characteristics

Nine doctoral nursing students participated in the study. The sample comprised six women and three men and represented varied age categories, experience profiles, and academic/professional backgrounds. Participant characteristics are summarized in Table 1.

### 3.2. Thematic Findings

Participant quotations are identified using codes P01–P09 to protect confidentiality. Analysis of the nine interviews generated four interrelated themes, summarized in Table 2. Together, these themes indicate that participants did not describe digital health readiness as a fixed individual attribute or as mere willingness to use technology. Instead, readiness was shaped by familiarity, perceived usefulness, system credibility, organizational conditions, and the extent to which digital technologies were perceived to be compatible with nursing work.

**Theme 1. Readiness was positive but conditional**

As summarized in Table 2, participants generally expressed a favorable orientation toward health technologies; however, their readiness remained conditional and depended on familiarity, workflow fit, and confidence that digital systems would support nursing work. Across the interviews, readiness ranged from openness and flexibility to a stronger sense that digital adoption had become part of contemporary nursing practice. Participants also emphasized that readiness depended on familiarity, workflow fit, and confidence that digital systems would support rather than undermine core nursing competence.

**Subtheme 1.1 From openness to professional necessity**

Some participants described readiness as openness to digital transformation. P01 described herself as “ready” and as having the flexibility to adapt to digital change, while P02 described herself as “to some extent” ready, reflecting a positive yet measured readiness. Other participants framed adoption as a professional expectation rather than a matter of personal preference. P09 stated:

“We have to adopt most of the programs used in practice.”

(P09)

These accounts suggest that readiness was not experienced as a single, stable position. For some participants, digital health was welcomed as a useful development; for others, it was increasingly understood as part of competent contemporary nursing practice.

**Subtheme 1.2 Familiarity and workflow fit**

Readiness was also strengthened through familiarity and fit with daily nursing work. Participants described digital systems as more acceptable when they were used repeatedly, became part of routine practice, and aligned with actual nursing tasks. P08 clearly captured this process:

“Once people get used to it and know how to deal with it, it becomes a permanent way of working.”

(P08)

P01 similarly linked rapid uptake to repeated use, follow-up, and a shift from paper-based work to electronic systems. These accounts indicate that readiness is developed through repeated use and practical integration rather than being fully formed before implementation.

Simultaneously, the participants did not endorse digitalization uncritically. P03 provided an important divergent account, explaining that digital systems reduced documentation delays but could also weaken manual or foundational skills among newer nurses if these skills were not maintained:

“It is good because it reduced delays… but it is not good for the new generation.”

(P03)

This reservation shows why the readiness was conditional. Digital tools were valued when they improved work, but participants remained cautious when technology was perceived as potentially replacing essential professional knowledge or manual competence. In relation to UTAUT2, this theme reflected effort expectancy, habit, and facilitating conditions through participants’ emphasis on familiarity, repeated use, workflow fit, and practical support; however, it also extended the framework by showing that readiness was interpreted as a professional judgment about whether digital technologies supported nursing work without weakening foundational competence.

**Theme 2. Adoption depended on practical value and system credibility**

As summarized in Table 2, participants evaluated digital health technologies based on two closely related considerations: whether the technology offered practical value in everyday use and whether it was credible enough to rely upon. Digital tools were valued when they improved access, reduced effort, enhanced efficiency, supported documentation, and contributed to safer care. However, usefulness alone was not sufficient. Participants also emphasized accuracy, privacy, and reliability as prerequisites for adoption.

**Subtheme 2.1 Access, convenience, and practical value**

A recurring source of value was easier access to services and information. Participants described digital health technologies as saving time, reducing travel, minimizing repeated visits, and supporting continuity of access to care and information across locations. P07 summarized this practical value as follows:

“All the services I need, I can get through my phone.”

(P07)

P02 described how digitally linked patient information allowed follow-up across different regions, while P06 emphasized the value of reviewing results, changing appointments, and accessing virtual services as a healthcare user. These accounts indicate that practical value was experienced in terms of time and place: digital health made care and information easier to access.

**Subtheme 2.2 Efficiency, safety, and care quality**

Participants also linked digital systems to efficiency, documentation integrity, communication, and safety. P05 highlighted the safety value of electronic documentation by contrasting it with handwritten records:

“There is no room for an error resulting from illegible handwriting.”

(P05)

P08 valued electronic records because they preserved documentation and allowed information to be revisited when necessary. P09 contrasted with earlier paper-based charting, which could take 1 to 3 h, with electronic documentation that could be completed in 20 to 30 min. Across these accounts, digital systems were valued not only for their speed but also for supporting clearer, safer, and more reliable care processes.

**Subtheme 2.3 Accuracy, privacy, and reliability**

Practical value did not guarantee adoption unless the system was trusted. Participants linked their willingness to use digital systems to their confidence in accuracy, reliability, documentation integrity, and protection of patient information. P09 expressed this clearly:

“If I am sure the program gives me accurate information… I will use it immediately.” (P09)Privacy and confidentiality are also important concerns. P07 described privacy as an immediate barrier for sensitive health topics, stating, “No matter how much technology develops, there remains fear about data privacy… especially when discussing sensitive topics.” P04 similarly linked acceptance to system reliability and trust in the authority behind technology. These accounts indicate that credibility functioned as a threshold condition for adoption: technologies had to be perceived as accurate, secure, and dependable before participants were willing to rely on them.

In relation to UTAUT2, this theme primarily reflected performance expectancy and effort expectancy through participants’ emphasis on usefulness, access, efficiency, and ease of use; however, it also extended the framework by showing that system credibility, including accuracy, privacy, reliability, and documentation integrity, functioned as a threshold condition for adoption.

**Theme 3. Adoption was organizationally mediated**

As summarized in Table 2, the participants did not describe digital health adoption as a purely individual decision. Instead, they portrayed adoption as being shaped by organizational and educational conditions. Leadership, peer culture, infrastructure, implementation quality, training, follow-up, and academic preparation all influenced whether readiness could be translated into practice.

**Subtheme 3.1 Leadership, peers, and organizational influence**

Leadership and institutional signaling were central to the participants’ accounts. P04 directly summarized this relationship:

“The institution comes first, and then the practitioner.”

(P04)

Participants described organizations as shaping whether digital tools were introduced, explained, accepted, and normalized in practice. Peer culture also influenced adoption. P02 noted that resistance sometimes stemmed more from colleagues than from leaders, while P06 described the role of emails, workshops, and prior announcements in encouraging readiness for adoption. P09 further emphasized the organization as a gatekeeper for whether a useful digital tool could reach practice, stating, “If the organization itself is not supportive of these technologies, how will I convince the nurses?” Across these accounts, adoption was socially and institutionally mediated through communication, endorsement, organizational decision-making, and workplace culture surrounding digital change.

**Subtheme 3.2 Infrastructure and implementation conditions**

Participants also emphasized that adoption depends on practical implementation conditions. Positive attitudes toward digital health can be undermined by limited device availability, inconsistent systems, poor accessibility, or insufficient implementation readiness in the work environment. P08 stated:

“If the devices are available and immediately accessible, that supports adoption.”

(P08)

P03 described situations in which several staff members had to share a limited number of devices, while P02 pointed to the difficulty of moving between hospitals that used different systems to store patient data. These examples show that infrastructure was not merely a background condition; rather, it directly shaped the effective use of digital health technologies in practice.

**Subtheme 3.3 Training, follow-up, and educational preparation**

Training was described as necessary but insufficient if it was delivered only once. Participants viewed digital capability as something that needed to be developed over time through practical preparation, ongoing reinforcement, and follow-up after its implementation. P03 emphasized that newly implemented systems require continued monitoring:

“Needs monitoring for three or six months.”

(P03)

Participants also located digital readiness before workplace implementation, particularly in terms of academic preparation. P01 emphasized the role of universities:

“The university is the one that plants the seed.”

(P01)

This subtheme shows that participants viewed readiness as developed rather than assumed. These accounts suggest that digital capability was shaped by formal education, workplace training, practical exposure, and continued support after implementation.

In relation to UTAUT2, this theme primarily reflected facilitating conditions and social influence, as evidenced by participants’ emphasis on leadership, peer culture, infrastructure, device availability, training, follow-up, and academic preparation. It also extended the framework by showing that organizational decision-making and implementation readiness shaped whether individual readiness could be translated into practice.

**Theme 4. Digital health was understood as supporting, not substituting for, nursing work**

As summarized in Table 2, this theme captured the professional meaning that participants attached to digital health technologies. Participants did not describe technology as replacing nurses. Instead, they described it as reducing avoidable burden, supporting documentation and communication, improving workflow, and allowing nurses to focus more on direct care while preserving the human and judgment-based core of nursing practice.

**Subtheme 4.1 Redirecting time toward direct care**

Participants did not describe reduced documentation time only as an efficiency gain. Rather, they connected it to the possibility of redirecting nursing time toward patient-facing activities, including direct care, patient education, monitoring, and follow-up. P07 explained:

“Instead of spending 5 or 10 -minutes writing notes for one patient, I can now finish it in one and a half or two minutes.”

(P07)

This account shows that participants valued digital health not simply because it reduced work but because it created more space for clinically meaningful nursing activities.

**Subtheme 4.2 Augmentation rather than replacement**

Participants consistently maintained that digital technologies and AI could support nursing work but could not replace the relational, communicative, and judgment-based dimensions of the profession. P06 expressed this boundary clearly:

“The presence of technology does not eliminate the need for a human being to talk with you.”

(P06)

P09 similarly emphasized support rather than replacement:

“We will not replace nurses with these applications or programs; rather, we can support them.”

(P09)

These accounts show that participants described technology as an assistant to nursing rather than as a substitute for nurses. Digital tools were viewed as useful for communication, documentation, monitoring, alerts, and evidence-based practice, but participants continued to position the nurse’s human presence, clinical judgment, and patient interaction as central to care.

In relation to UTAUT2, this theme reflected performance expectancy through participants’ emphasis on reduced documentation burden, improved workflow, communication, monitoring, and support for evidence-informed practices. However, it also extended the framework by showing that acceptability depended on whether digital technologies preserved the relational, communicative, and judgment-based dimensions of nursing care.

## 4. Discussion

This study explored doctoral nursing students’ perceptions of readiness to adopt digital health technologies in Saudi Arabia. UTAUT2 guided the interpretation of the findings without predetermining them. Readiness was not described as a simple willingness to use technology, but as a professional judgment shaped by familiarity, practical value, system credibility, organizational support, and fit with nursing work. Across the four themes, readiness was positive but conditional; adoption required practical value and system credibility; implementation depended on organizational and educational conditions; and digital health was viewed as supporting, not substituting for, nursing work. This framing extends technology acceptance perspectives by showing that, for doctoral nursing students, readiness was not only an individual acceptance process but also a professional, relational, and organizationally mediated judgment within the context of Saudi healthcare transformation.

### 4.1. Interpreting the Findings Through UTAUT2

UTAUT2 helped explain why participants were more receptive to digital health when technologies were useful, easy to use, supported by the organization, familiar through repeated use, and increasingly expected in contemporary nursing practice. These patterns are consistent with UTAUT2, particularly performance expectancy, effort expectancy, social influence, facilitating conditions, experience, and habit [20]. However, the participants did not evaluate their readiness only in terms of their intention to use the technology. They also judged whether digital systems were accurate, trustworthy, professionally appropriate, and compatible with nursing competence and everyday practice.

Therefore, UTAUT2 was useful for identifying adoption-related conditions, but it had limitations as a stand-alone interpretive lens. Its constructs helped explain why usefulness, ease of use, repeated experience, social influence, and facilitating conditions mattered; however, they did not fully capture the relational, professional, and ethical meanings that the participants attached to nursing work. Participants’ concerns about preserving human communication, clinical judgment, privacy, documentation integrity, and foundational competence showed that readiness involved evaluating whether digital technologies aligned with the values and responsibilities of nursing. In this sense, the qualitative findings extend UTAUT2 by positioning readiness as a context-sensitive professional judgment rather than as merely an intention to use technology. 

### 4.2. Readiness Was Positive but Conditional

In this study, readiness was best understood as a conditional professional stance rather than general enthusiasm for digital health. Participants were broadly receptive to digital transformation and, in some cases, framed adoption as part of contemporary nursing practice. However, this readiness was strengthened only when digital technologies became familiar, useful, aligned with everyday nursing workflows, and perceived as supporting nursing competence. This pattern aligns with the UTAUT2 constructs of performance expectancy, effort expectancy, social influence, facilitating conditions, experience, and habit [20]. However, the participants’ accounts also moved beyond mere technology acceptance. They evaluated digital health through a nursing lens, asking whether the systems were workable and compatible with nursing’s knowledge, skills, and responsibilities.

This interpretation is consistent with broader evidence suggesting that favorable attitudes toward digital health may not lead to meaningful adoption unless practical barriers such as infrastructure, workload, training, and system integration are addressed [3]. Nursing-focused evidence similarly emphasizes that digital capability depends on user proficiency, access to relevant information at the point of care, fit-for-purpose systems, and investment in professional development [5]. At the same time, participants’ concerns about foundational or manual competence add an important caution. These concerns should not be interpreted as resistance to technology but as a professional expectation that digital systems should support rather than undermine nursing competence. This aligns with Hants et al. [30], who found that digital health systems do not always capture the full nursing process, particularly interventions and outcome evaluation. Overall, the participants’ caution did not contradict their readiness. Thus, the contribution of this theme is that conditional readiness was not simply hesitation toward technology; rather, it reflected a professional concern regarding maintaining nursing competence while adopting digital tools. 

### 4.3. Adoption Depended on Practical Value and System Credibility

Participants evaluated digital health technologies based on two linked concerns: practical value and system credibility. Digital tools were not valued simply because they were new; they became meaningful when they improved access to services and information, reduced effort and time, strengthened documentation, and supported safer care. Lin et al. [31] and Zha et al. [32] similarly reported that perceived usefulness and performance expectancy influence nurses’ and healthcare practitioners’ intentions to use digital systems. [31,32]. In the Saudi context, a broader study of healthcare practitioners found that performance expectancy predicted intention to use telehealth, supporting the relevance of perceived benefit in digital health adoption beyond this doctoral nursing student sample [17]. The present study adds nursing-focused qualitative depth by showing what practical value means to participants. easier access, continuity across locations, time saving, documentation integrity, and reduced risk of error. This interpretation is also consistent with national health transformation priorities around access, quality, efficiency, and integrated services, although this policy context should be used to situate the findings rather than as direct evidence of readiness [16].

At the same time, participants did not consider usefulness alone sufficient for adoption. Their willingness to rely on digital systems also depended on their confidence in accuracy, privacy protection, documentation integrity, and the reliability of the system or the authority responsible for it. This finding qualifies acceptance-oriented evidence: perceived usefulness may encourage intention, but adoption can still be limited when users question confidentiality, trust, or system dependability. Evidence on nurses’ personal smartphone use shows a similar tension: rapid access to information and communication may support practice, while concerns about distraction, privacy, patient perceptions, and policy ambiguity remain [13]. Evidence from NICU nursing settings also points to the role of trust and motivation in technology-mediated practice, although this evidence is contextual rather than directly comparable [33]. Overall, the participants did not consider practical value and system credibility as separate considerations. For them, digital health technologies had to be useful in everyday nursing work and sufficiently trustworthy to support their professional judgment. Thus, the contribution of this theme is not simply that usefulness mattered, but that usefulness was inseparable from credibility in the participants’ judgments of readiness.

### 4.4. Adoption Was Organizationally Mediated

Participants did not portray digital health adoption as a matter of individual willingness alone. They understood readiness as shaped by organizational and educational conditions, including leadership, peer norms, communication, infrastructure, system consistency, device access, training, and follow-up. This interpretation gives the UTAUT2 constructs of social influence and facilitating conditions a nursing-specific meaning: organizational support shaped whether participants’ readiness could be translated into everyday nursing practice. Burgess and Honey [11] emphasized the importance of leadership, training, and organizational support in digital health adoption, while Wosny et al. [7] and Alotaibi et al. [34] highlighted the importance of workflow integration and implementation.

In addition, as the participants were doctoral nursing students, organizational mediation also had an educational dimension. Their accounts positioned universities as early sites for developing digital readiness, while emphasizing that healthcare organizations were expected to sustain this readiness through practical training, system access, leadership, and post-implementation support. Han et al. [35] and Kulju et al. [9] similarly emphasized the role of education and support in developing digital competence, while Tischendorf et al. [10] noted limited evidence for well-evaluated continuing education programs for nursing digital competencies.

At the policy level, the findings can be situated within global and Saudi priorities regarding governance, workforce capability, interoperability, access, quality, efficiency, and digital solutions; however, these sources provide context rather than direct evidence of readiness [2,16]. Overall, this theme suggests that, within this sample, readiness became more meaningful when academic and clinical organizations created conditions that made digital health usable, supported, and integrated into nursing practices. 

### 4.5. Digital Health Was Understood as Supporting, Not Substituting for, Nursing Work

Participants drew a clear boundary between using technology to support nursing work and allowing it to substitute for nursing work. They welcomed digital tools when they made care more efficient, safer, or easier to coordinate, but they continued to position clinical judgment, communication, and relational presence as aspects of nursing work that should not be replaced by technology. This interpretation aligns with nursing literature suggesting that digital and AI-enabled technologies may reshape nursing roles and workflows without replacing nurses [36]. The present study extends this argument by suggesting that efficiency was interpreted through nursing values: time saved through documentation or communication was meaningful only when it could be redirected toward patient care.

However, this supportive view was conditional. Participants’ accounts suggest that digital health was perceived as supporting nursing when it reduced avoidable burdens without weakening clinical judgment or diminishing the visibility of nursing work. Schlicht et al. [37] reported that digital nursing technologies may support information management and job control while also contributing to workload, frustration, cognitive overload, stress, and burnout when poorly integrated. Wosny et al. [7] similarly highlighted the tensions in professionals’ experiences using digital tools. This finding also aligns with Hants et al. [30], who found that digital systems capture assessment and planning more comprehensively than interventions and outcome evaluations. Thus, for participants, the key issue was not whether digital tools should be used but whether they preserved nursing’s professional visibility and clinical contribution within digitally mediated care.

### 4.6. Implications and Contribution to Knowledge

The findings suggest that digital health readiness should be developed across the academic–practice continuum rather than assumed at the point of implementation. Within the limits of this small qualitative sample, nursing education and clinical organizations may benefit from moving beyond general awareness toward applied digital health preparation, including electronic documentation, telehealth communication, privacy and ethics awareness, simulation or practice-based exposure, workflow-aligned implementation, training, and post-implementation follow-ups. These implications should be understood as contextually grounded directions for nursing education and practice rather than broadly generalizable prescriptions.

This study contributes to the knowledge by offering a qualitative account of digital health readiness as conditional, professionally situated, and shaped by factors beyond mere intention to use technology. While technology-acceptance perspectives often emphasize behavioral intention and perceived usefulness, these findings show how doctoral nursing students interpreted readiness in terms of practical value, familiarity, system credibility, organizational support, and the preservation of nursing competence. The findings support the use of UTAUT2 as a sensitizing framework while also extending it by highlighting nursing-specific considerations, such as trust, privacy, reliability, workflow fit, foundational competence, and the human core of care.

### 4.7. Study Limitations

Several limitations should be considered when interpreting the findings of this study. The study was conducted with a small purposive volunteer sample of doctoral nursing students from one public university in Saudi Arabia, which may limit its transferability to other nursing populations, educational levels, and institutional contexts. Although 20 eligible students were invited and nine participated, reasons for non-response were not collected, and the findings reflect self-reported perceptions of readiness rather than direct observation of technology use or sustained adoption behavior. The interviews were relatively brief, and participant member checking of transcripts or themes was not conducted; however, credibility and transparency were supported through verbatim transcription, repeated transcript review, reflexive memoing, illustrative quotations, cross-case matrix comparisons, and documentation of analytic decisions. The first author’s position within the same academic context may have facilitated rapport and contextual understanding; therefore, reflexive memoing was used to consider how familiarity with the setting might influence data collection and interpretation.

### 4.8. Future Research Directions

Future research should examine digital health readiness across broader nursing groups and institutional contexts, including nursing students, clinical nurses, educators, and leaders. Comparative and longitudinal studies may help clarify how educational level, clinical experience, prior exposure to digital systems, and organizational context shape readiness over time and during the actual implementation. Future work could incorporate direct observation or system-use data to examine how perceived readiness translates into sustained digital health adoption in nursing practice.

## 5. Conclusions

This exploratory qualitative study provides insights into how a small sample of doctoral nursing students at a public university in Saudi Arabia understood their readiness to adopt digital health technologies. Participants viewed digital health positively, but their readiness was not simply a matter of willingness to use technology. Rather, readiness was interpreted as a professionally situated judgment shaped by practical value, familiarity, system credibility, organizational support, educational preparation, and the perceived fit between digital technologies and nursing work. The findings suggest that digital health readiness is a shared educational, organizational, technological, and professional responsibility. Within this context, digital transformation may be most meaningful when supported by credible systems, digitally capable nurses, and implementation conditions that strengthen practice while preserving the human, relational, and judgment-based core of nursing care.

## Figures and Tables

**Table 1:** Participant characteristics (N = 9).

Characteristic	Category	n/Summary
**Gender**	Female	6
	Male	3
**Age category**	25–30 years	2
	31–35 years	3
	36–40 years	2
	Above 40 years	2
**Experience profile**	Clinical/professional only	4
	Academic only	1
	Combined clinical/professional and academic	4
**Clinical/professional experience**	Range	0–23 years
**Academic experience**	Range among participants with academic experience	1 to more than 10 years

**Table 2:** Themes, subthemes, and main findings.

Theme	Subtheme	Main Finding
Theme 1. Readiness was positive but conditional	1.1 From openness to professional necessity	Readiness ranged from openness and flexibility to a stronger sense that digital adoption had become part of contemporary nursing practice.
	1.2 Familiarity and workflow fit	Readiness strengthened through repeated use, familiarity, and alignment with everyday nursing workflow, but remained conditional when technology was perceived as weakening foundational or manual competence.
Theme 2. Adoption depended on practical value and system credibility	2.1 Access, convenience, and practical value	Digital health was valued for improving access to services and information, saving time, reducing travel, and supporting continuity across locations.
	2.2 Efficiency, safety, and care quality	Digital systems were valued for improving documentation, communication, efficiency, and safer care processes.
	2.3 Accuracy, privacy, and reliability	Adoption depended on trust in system accuracy, reliability, documentation integrity, and protection of patient information.
Theme 3. Adoption was organizationally mediated	3.1 Leadership, peers, and organizational influence	Leadership, institutional communication, peer culture, and workplace norms shaped whether digital tools were accepted and normalized.
	3.2 Infrastructure and implementation conditions	Device availability, system consistency, accessibility, and implementation readiness determined whether digital tools could be used effectively.
	3.3 Training, follow-up, and educational preparation	Readiness was developed through academic preparation, practical training, ongoing reinforcement, and post-implementation follow-up.
Theme 4. Digital health was understood as supporting, not substituting for, nursing work	4.1 Redirecting time toward direct care	Digital systems reduced documentation burden and created more space for direct care, patient education, monitoring, and follow-up.
	4.2 Augmentation rather than replacement	Participants viewed digital technologies and AI as supporting nursing work while preserving human presence, communication, and clinical judgment.

*Note*. Themes and subthemes were generated through a hybrid deductive–inductive thematic analysis. UTAUT2 informed the deductive component of the analysis but did not predetermine the final theme structure.

## Data Availability

The interview data are not publicly available due to participant confidentiality and ethical restrictions. Relevant de-identified excerpts supporting the findings are included in the article.

## Supplementary Materials

The following supporting information can be downloaded at: https://www.mdpi.com/article/10.3390/healthcare14111594/s1, File S1: Completed COREQ checklist; File S2: Interview guide with follow-up probes and UTAUT2 mapping.
