# Supplementary material for: Digital Health Technology Adoption Readiness Among Doctoral Nursing Students in Saudi Arabia: An Exploratory Qualitative Study
Source: Healthcare (Basel). 2026 Jun 5;14(11):1594. doi: 10.3390/healthcare14111594 (PMC13256542; doi:10.3390/healthcare14111594)
Supplement: Supplementary file 1 [file healthcare-14-01594-s001.zip › Supplementary File S1.pdf]

COREQ (Consolidated Criteria for Reporting Qualitative Research) Checklist

A checklist of items that should be included in reports of qualitative research. You must report the page number in your manuscript where you consider each of the items listed in this checklist. If you have not included this information, either revise your manuscript accordingly before submitting or note N/A.

Topic	Item No.	Guide Questions/Description	Reported on Page No.
**Domain 1: Research team and reflexivity**			
Personal characteristics			
Interviewer/facilitator	1	Which author/s conducted the interview or focus group?	p. 6, line 251; p. 7, lines 283-285
Credentials	2	What were the researcher's credentials? E.g., PhD, MD	p. 8, lines 333–336
Occupation	3	What was their occupation at the time of the study?	p. 8, lines 333–336
Gender	4	Was the researcher male or female?	p. 8, lines 333–336
Experience and training	5	What experience or training did the researcher have?	p. 8, lines 333–336; p. 7, lines 290–320
Relationship with participants			
Relationship established	6	Was a relationship established prior to study commencement?	p. 5, lines 206-210; p. 8, lines 333-338
Participant's knowledge of the interviewer	7	What did the participants know about the researcher? e.g., personal goals, reasons for doing the research	p. 5, lines 206-210; p. 6, lines 260-263; p. 8, lines 333-338
Interviewer characteristics	8	What characteristics were reported about the interviewer/facilitator? e.g., bias, assumptions, reasons, and interests in the research topic	p. 8, lines 333–348
**Domain 2: Study design**			
Theoretical framework			
Methodological orientation and Theory	9	What methodological orientation was stated to underpin the study? e.g., grounded theory, discourse analysis, ethnography, phenomenology, content analysis	p. 5, lines 180-191
Participant selection			
Sampling	10	How were participants selected? e.g., purposive, convenience, consecutive, snowball	p. 5, lines 193-198
Method of approach	11	How were participants approached? e.g., face-to-face, telephone, mail, email	p. 5, lines 199-202
Sample size	12	How many participants were in the study?	p. 5, lines 201-204; p. 5, lines 220-222
Non-participation	13	How many people refused to participate or dropped out? Reasons?	p. 5, lines 201-205; p. 19, lines 810-815
Setting			
Setting of data collection	14	Where was the data collected? e.g., home, clinic, workplace	p. 5, lines 193-195; p. 6, lines 251-254
Presence of non-	15	Was anyone else present besides the participants and researchers?	p. 6, lines 254-255

Topic	Item No.	Guide Questions/Description	Reported on Page No.
participants			
Description of sample	16	What are the important characteristics of the sample? e.g., demographic data, date	p. 8, lines 363-367; p. 9, Table 1
Data collection			
Interview guide	17	Were questions, prompts, and guides provided by the authors? Was it pilot tested?	p. 6, lines 271-275; p. 7, lines 276-281; Supplementary File S2
Repeat interviews	18	Were repeat interviews carried out? If yes, how many?	p. 6, line 253
Audio/visual recording	19	Did the research use audio or visual recording to collect the data?	p. 6, lines 260-261; p. 7, lines 283-284
Field notes	20	Were field notes made during and/or after the interview or focus group?	p. 7, lines 286-287
Duration	21	What was the duration of the interviews or focus group?	p. 6, lines 253-254
Data saturation	22	Was data saturation discussed?	p. 5, lines 223-227; p. 6, lines 228-234
Transcripts returned	23	Were transcripts returned to participants for comment and/or correction?	p. 8, lines 341-344
**Domain 3: analysis and findings**			
Data analysis			
Number of data coders	24	How many data coders coded the data?	p. 7, lines 306-308
Description of the coding tree	25	Did the authors provide a description of the coding tree?	p. 7, lines 306-317
Derivation of themes	26	Were themes identified in advance or derived from the data?	p. 7, lines 290-299; p. 7, lines 312-317
Software	27	What software, if applicable, was used to manage the data?	p. 7, lines 306-308
Participant checking	28	Did participants provide feedback on the findings?	p. 8, lines 341-344; p. 19, lines 816-820
Reporting			
Quotations presented	29	Were participant quotations presented to illustrate the themes/findings? Was each quotation identified? e.g., participant number	p. 9, line 370; pp. 10-14, lines 382-589
Data and findings consistent	30	Was there consistency between the data presented and the findings?	p. 9, lines 371-379; pp. 9-10, Table 2; pp. 10-14, lines 381-589
Clarity of major themes	31	Were major themes clearly presented in the findings?	pp. 9-10, Table 2; p. 10, line 381; p. 11, line 429; p. 12, line 482; p. 14, line 549
Clarity of minor themes	32	Is there a description of diverse cases or a discussion of minor themes?	pp. 9-10, Table 2; pp. 10-14, lines 391-589; divergent account on p. 11, lines 415-420

Developed from: Tong A, Sainsbury P, Craig J. Consolidated criteria for reporting qualitative research (COREQ): a 32-item checklist for interviews and focus groups. *International Journal for Quality in Health Care*. 2007. Volume 19, Number 6: pp. 349-357

Once you have completed this checklist, please save a copy and upload it with your submission. DO NOT include this checklist as part of the main manuscript document. It must be uploaded as a separate file.
