# Supplementary material for: Digital Health Technology Adoption Readiness Among Doctoral Nursing Students in Saudi Arabia: An Exploratory Qualitative Study
Source: Healthcare (Basel). 2026 Jun 5;14(11):1594. doi: 10.3390/healthcare14111594 (PMC13256542; doi:10.3390/healthcare14111594)
Supplement: Supplementary file 1 [file healthcare-14-01594-s001.zip › Supplementary File S2-Interview Guide UTAUT2 Mapping.pdf]

Supplementary File S2. Interview Guide with Selected Follow-up Probes and UTAUT2 Mapping

Digital Health Technology Adoption Readiness among Doctoral Nursing Students in Saudi Arabia: An Exploratory Qualitative Study

Note. The interviews were conducted in Arabic. This supplementary file presents the English reporting version of the interview guide and selected follow-up probes used to support clarification and depth during the interviews. Probes were used flexibly according to participants' responses and were not necessarily asked in the same sequence or in full for every participant.

Demographic prompts

- Age
- Gender
- Years of academic and/or professional experience

Table S2.1. Interview guide with selected follow-up probes

No.	Main interview question	Selected follow-up probes	Primary focus
1	How would you describe your level of readiness to adopt digital health technologies in the field of nursing?	• What do you mean by "readiness"?<br>• Can you provide an example that illustrates this readiness?<br>• Have you had an experience that influenced your readiness?<br>• What might increase or reduce this readiness?	Perceived readiness; familiarity; prior experience; openness or conditional readiness
2	What factors encourage you to use digital health technologies, such as perceived benefits or ease of use?	• What benefits do you see in these technologies?<br>• Do they make your work easier? If so, how?<br>• Can you describe a situation in which technology was particularly useful?<br>• Do colleagues, supervisors, or leaders influence your use of these technologies?<br>• What makes you motivated to use them?	Perceived usefulness; ease of use; performance expectancy; effort expectancy; social influence
3	What challenges or barriers might influence your readiness to adopt digital health technologies?	• Have you faced difficulty using any digital system?<br>• Was the training sufficient?<br>• Does workload affect the use of technology?<br>• Do you have concerns about privacy, errors, or system complexity?<br>• What might make technology difficult to use?	Barriers; training; workload; privacy; system credibility; facilitating conditions
4	What role does the environment or organization you belong to play in supporting or hindering your readiness to adopt digital health technologies?	• Does your organization provide adequate training?<br>• Is technical support available when needed?<br>• How do leaders or supervisors influence technology use?<br>• Does the organizational culture encourage digital innovation?<br>• What type of support is most needed?	Organizational support; leadership; peer culture; facilitating conditions; social influence

No.	Main interview question	Selected follow-up probes	Primary focus
5	From your perspective, how can digital health technologies contribute to advancing the nursing field or improving nursing practice in the future?	• Could technology improve the quality of care?<br>• How might it affect work efficiency?<br>• Do you think it will change the future role of nurses?<br>• What would you like to see in the future of digital nursing?<br>• Are there particular technologies that you see as promising?	Future nursing practice; augmentation rather than replacement; professional role; direct care; clinical judgment

Table S2.2. UTAUT2 mapping across interview guide, coding, and final themes

UTAUT2 construct / analytic category	Interview guide alignment	Coding process alignment	Final theme linkage
Performance expectancy	Questions 2 and 5 explored perceived benefits, usefulness, quality of care, efficiency, and future contributions to nursing practice.	Codes included perceived usefulness, access convenience, documentation efficiency, safety, quality of care, and reduced burden.	Theme 2: Practical value and system credibility; Theme 4: Supporting, not substituting for, nursing work.
Effort expectancy	Questions 2 and 3 explored ease of use, system complexity, and difficulty using digital systems.	Codes included ease of use, system complexity, usability barriers, and workflow fit.	Theme 1.2: Familiarity and workflow fit; Theme 2: Practical value in everyday use.
Social influence	Questions 2 and 4 explored peer influence, supervisor influence, leadership, and organizational expectations.	Codes included peer influence, leadership support, organizational signaling, and workplace norms.	Theme 3: Adoption was organizationally mediated.
Facilitating conditions	Questions 3 and 4 explored training, technical support, infrastructure, device availability, workload, and organizational readiness.	Codes included training need, technical support, infrastructure/device availability, system consistency, and implementation conditions.	Theme 3.2: Infrastructure and implementation conditions; Theme 3.3: Training, follow-up, and educational preparation.
Experience and habit	Questions 1 and 2 explored readiness, prior exposure, repeated use, and familiarity with digital systems.	Codes included familiarity, repeated use, routine integration, and practical exposure.	Theme 1: Readiness was positive but conditional.
Behavioral intention/readiness	Question 1 directly explored perceived readiness to adopt digital health technologies.	Codes included perceived readiness, willingness to use, conditional acceptance, and professional necessity.	Theme 1.1: From openness to professional necessity.
Price value	Not emphasized because participants discussed digital health primarily as students, academic/clinical users, and professionals within institutional systems rather than as purchasers of technologies.	Price value was not a prominent code in the data.	Not prominent in the final themes.
Hedonic motivation	Not emphasized because the study focused on professional readiness, nursing work, and clinical/educational adoption rather than enjoyment of technology use.	Hedonic motivation was not a prominent code in the data.	Not prominent in the final themes.
Emergent nursing-specific considerations beyond UTAUT2	Questions 3–5 allowed participants to discuss concerns and meanings beyond predefined UTAUT2 constructs.	Emergent codes included privacy, system credibility, documentation integrity, foundational/manual competence, human presence, and nursing judgment.	Themes 2 and 4 extended UTAUT2 by foregrounding trust, reliability, privacy, foundational competence, and the human core of nursing care.

Abbreviation. UTAUT2 = Unified Theory of Acceptance and Use of Technology 2.
